# Supplementary material for: Interaction of Trichoderma Species with Fusarium graminearum Growth and Its Trichothecene Biosynthesis as Further Contribution in Selection of Potential Biocontrol Agents
Source: J Fungi (Basel). 2025 Jul 14;11(7):521. doi: 10.3390/jof11070521 (PMC12295510; doi:10.3390/jof11070521)
Supplement: Supplementary file 1 [file jof-11-00521-s001.zip › jof-3723859-supplementary.pdf]

Multimodal interactions of toxigenic *Fusarium graminearum* and deoxynivalenol with *Trichoderma* indicate potential effects of *Trichoderma* as a microbial agent.

Xianfeng Ren [a, c], Guidong Li [a,d], Lixia Fan [a, c], Bingchun Zhang [a, c], Mingxiao Ning [a, c], Jing Gao [b], Fei Wang [b], Changying Guo [a, c] *, Antonio F. Logrieco [b]*

a Institute of Agricultural Quality Standards and Testing Technology of Shandong Academy of Agricultural Sciences, Jinan, China

b Xianghu Laboratory (Zhejiang Agricultural Laboratory), Hangzhou, China

c Shandong Provincial Key Laboratory of Test Technology on Food Quality and Safety, Jinan, China

d Henan University of Science and Technology, College of Food and Bioengineering, Luoyang, China

[*] Corresponding author:

Changying Guo (Guo C.); Emai: cyguo808@163.com; Tel: +86 80 66657924;

Antonio F. Logrieco (Antonio F.L.); Emai: antonio.logrieco@ispa.cnr.it; Tel.: ???????;

Table S1. Strains of *Trichoderma* spp. and *F. graminearum* used in this study.

**Species** [(a)]	**Strain** [(b)]	**Geographical Origin**	**Source**
T. asperellum	GC-T2	Tibet, China	Soil
	GC-T4	Tibet, China	Soil
T. atroviride	GC-T78	Yunnan, China	Rotten wood in soil
	GC-T8	Tibet, China	Soil
	GC-T9	Tibet, China	Soil
T. citrinoviride	GC-T19	Tibet, China	Soil
	GC-T20	Tibet, China	Soil
	GC-T21	Tibet, China	Soil
T. dorotheae	GC-T24	Tibet, China	Soil
	GC-T25	Tibet, China	Soil
T. erinaceum	GC-T27	Tibet, China	Soil
T. gamsii	GC-T26	Tibet, China	Soil
T. harzianum	GC-T76-1	Yunnan, China	Rotten wood soil
	GC-T40	Shandong, China	Plant debris in soil
	GC-T40-1	Tibet, China	Soil
	GC-T41	Tibet, China	Soil
T. hispanicum	GC-T42	Tibet, China	Soil
T. inhamatum	GC-T44	Tibet, China	Soil
T. koningii	GC-T75-1	Yunnan, China	Rotten wood in soil
T. longifialidicum	GC-T87	Tibet, China	Soil
T. reesei	GC-T18-1	China	Rotten wood in soil
T. sulphureum	GC-T43-1	China	Rotten wood in soil
T. velutinum	GC-T82	Tibet, China	Soil
	GC-T83	Tibet, China	Soil
	GC-T85	Tibet, China	Soil
T. viride	GC-T45-1	Shandong, China	Soil
T. virilente	GC-T88	Tibet, China	Soil
	GC-T89	Tibet, China	Soil
F. graminearum	PG-Fg1	Jinan, China	Wheat

[(a)]Species identified by sequence analysis of ITS-1 and ITS-2.[(b)]Strains were named by our laboratory.

Table S2. Primers used in this study.

Gene	Primer Code	Sequence (5′–3′)	Reference
β-tubulin	Tub_F	GGTCGTTACCTCACCTGCTCT	[1]
	Tub_R	GGATGTTGCGCATCTGGT	
tri4	Tri4_F	CTTGATGGAGCCTTCTCAGC	[2]
	Tri4_R	CATCAAGATAGTCCTTATGTTC	
tri5	Tri5_F	TCTATGGCCCAAGGACCTGTTTGA	[3]
	Tri5_R	TGACCCAAACCATCCAGTTCTCCA	
tri6	Tri6_F	CGTGCTGACGTGGTTCGAGTGC	[2]
	Tri6_R	CTATGGAATGGGTCGGCGAATC	
tri10	Tri10_F	CGCTCTCATATGAGTACGTTGGC	[2]
	Tri10_R	CCATGAATGGTGAAGATGGGC	

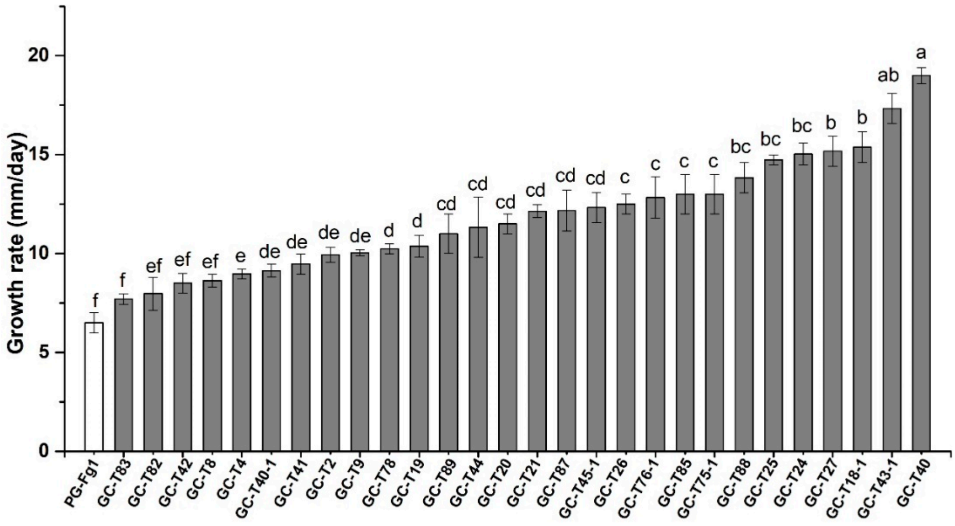

Figure S1. Average radial growth rate (mm/day) of *F. graminearum*PG-Fg1 and *Trichoderma* isolates. Values are means ± SD (*n* = 3).

1. Ren, X., et al., *Potential of Trichoderma spp. for Biocontrol of Aflatoxin-Producing Aspergillus flavus.* Toxins (Basel), 2022. **14**(2).
2. Tijerino, A., et al., *Overexpression of the Trichoderma brevicompactum tri5 gene: effect on the expression of the trichodermin biosynthetic genes and on tomato seedlings.* Toxins (Basel), 2011. **3**(9): p. 1220-32.
3. Bolanos-Carriel, C., et al., *Tri5gene expression analysis during postharvest storage of wheat grain from field plots treated with a triazole and a strobilurin fungicide.* Canadian Journal of Plant Pathology, 2020. **42**(4): p. 547-559.
